# Dissemination of Aerosol and Splatter in Clinical Environment during Cavity Preparation: An In Vitro Study

**DOI:** 10.3390/ijerph18073773

**Published:** 2021-04-04

**Authors:** Muhammad Adeel Ahmed, Rizwan Jouhar

**Affiliations:** Department of Restorative Dentistry and Endodontics, College of Dentistry, King Faisal University, Al-Ahsa 31982, Saudi Arabia; rjouhar@kfu.edu.sa

**Keywords:** dental personnel, aerosol, splatter, infection control, clinical environment, COVID-19

## Abstract

Dental health care workers around the world are in a constant state of fear and anxiety because they work in a constrained space of the dental practice. During routine dental procedures, they are exposed to aerosol and splatter. These airborne particles pose a great risk of transmitting contagious infections to health care workers and patients, especially in an era of social distancing due to COVID-19. The current study was conducted to evaluate contamination amount, duration, the distance of aerosol, and splatter produced after cavity preparation using a two-hole and four-hole handpiece. The study was performed on a dental manikin in a dental simulation laboratory at the College of Dentistry, King Faisal University Al Ahsa. The dental manikin was set to a reclined position to simulate the clinical operatory position of the patient for dental restorative procedures. Aerosol and splatter were collected on Grade 1 qualitative cotton cellulose filter paper. These were placed on adhesive tape extending from the headrest of the dental manikin in six different directions (2, 4, 6, 8, 10, and 12 o’clock) for up to 60 inches and on certain positions of the operator and assistant such as the chest, head, forearms, upper leg, and inside facemask. Class V cavity preparation was done by the principal investigator at a specific time of 3 min on tooth #11 using a two-hole high-speed handpiece, then on the next day, Class V cavity preparation was performed on tooth #21 by a four-hole handpiece. High volume suction was used throughout the cavity preparation. Immediately after cavity preparation, the first filter paper disc was replaced with new ones in all positions. The second set of filter papers was removed after 30 min. Transparent grids were used to count the contamination area on the filter paper disc. No statistically significant difference was found in the mean amount of aerosol and splatter produced by both handpieces, however, a statistically significant difference was found in an amount of aerosol and splatter produced at a 12, 24, and 36 inches distance immediately after cavity preparation and 30 min after cavity preparation, regardless of the type of handpiece used. It is advisable to refrain from removing the personal protective barriers immediately after the procedure within the vicinity of the dental practice. The use of other adjuncts such as high volume suction to reduce the spread of aerosol and splatter is also recommended.

## 1. Introduction

The majority of the dental health care workers are working in the constrained space of dental practice [1]. During routine dental procedures, they are exposed to aerosol and splatter [2]. Aerosol is defined as very tiny particles of less than 50 micrometers in diameter and has the potential ability to remain suspended in the air for a considerable time until they settle on the environmental surface or enter into the respiratory tract [3]. On the other hand, splatter is larger particles of more than 50 micrometers in diameter and is believed to stay in the air for a short time due to its size [4]. These airborne particles pose a great risk of carrying and transmitting contagious infections to health care workers and patients. Several diseases such as tuberculosis, measles, severe acute respiratory syndrome, and herpetic viral infection have been reported to be transmitted through the airborne route [2,5]. Studies have shown concern that the highly contaminated breathing zone in a dental practice could be the reason for the increased prevalence of the respiratory disease among dentists [5,6].

Dental procedures involving the use of a high- or low-speed handpiece, ultrasonic scaler, and air-water simultaneous spray from a triple syringe are liable to produce a huge amount of aerosols in the dental environment contaminated with saliva and/or blood [7,8]. A high-speed handpiece for cavity preparation is usually available as two-hole and three or four-hole. A two-hole high-speed handpiece is designed to have one hole for water flow and the other for airflow. In this handpiece, the bur continues to rotate even after the clinician removes their foot from the foot pedal, while in the three-hole handpiece, the third hole serves as a vent and allows excessive air collected in the head of the handpiece to exit, thus stopping the bur earlier than the two-hole handpiece. Similarly, the four-hole handpiece is designed to have an additional hole for fiber optics [9]. Presumably, it is a common finding that clinicians usually withdraw the handpiece from the mouth once the foot pedal is released, especially when working on the labial side of anterior teeth to avoid accidental tissue damage [10]. If the bur is moving outside the mouth, as in the case of the two-hole handpiece, there are likely chances that it will generate more aerosol in the air.

Dental rubber dam isolation reduces microbial contamination by controlling the dissemination of aerosol and splatter in clinical ambiance. Studies have also highlighted the significant role of high volume suction in mitigating the production of aerosol and splatter generated by the high-speed handpiece during dental procedures to more than 90% [11,12].

Recently, the airborne route of infection has received great attention due to the COVID-19 pandemic [13]. The impact of the COVID-19 pandemic entails the Centers for Disease Control and Prevention (CDC) and American Dental Association (ADA) to change their guidelines for cross infection control in dentistry (source: ADA Coronavirus (COVID-19) Center for Dentists). Fear and anxiety among dentists prevailed during the COVID-19 pandemic. Several studies have reported that dentists around the globe are reluctant to perform routine dental procedures due to psychological distress, lack of coordination between health care services, the emergence of new variants of COVID-19, and fear of acquiring and transmitting the infection to their family [14,15,16,17,18,19]. Working in dental (teaching) hospitals with multiple operators, staff and patients pose a higher risk of acquiring COVID-19 infection. The paucity of strong clinical evidence on aerosol and splatter contamination distance, duration may be a barrier for the implementation of quality dental services and dental education, which in turn are likely to influence the quality of care provided to patients and affect the learning of students, if not addressed promptly.

Considering the aforementioned rationale, the current study was conducted to evaluate contamination amount, duration, the distance of aerosol, and splatter produced after the cavity preparation using a two-hole and four-hole high-speed handpiece. In addition, it may help clinicians to propose guidelines to design adequate space for safe dental operations.

## 2. Materials and Methods

**Figure 1:**
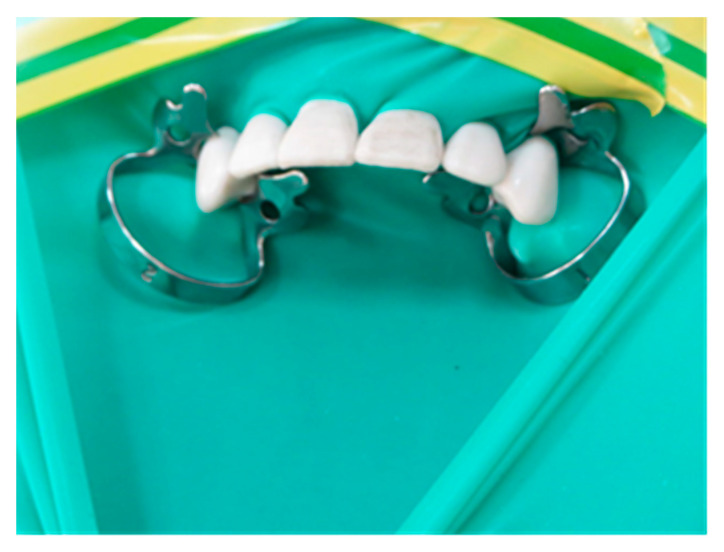
Upper anterior quadrant isolation by the rubber dam.

**Figure 2:**
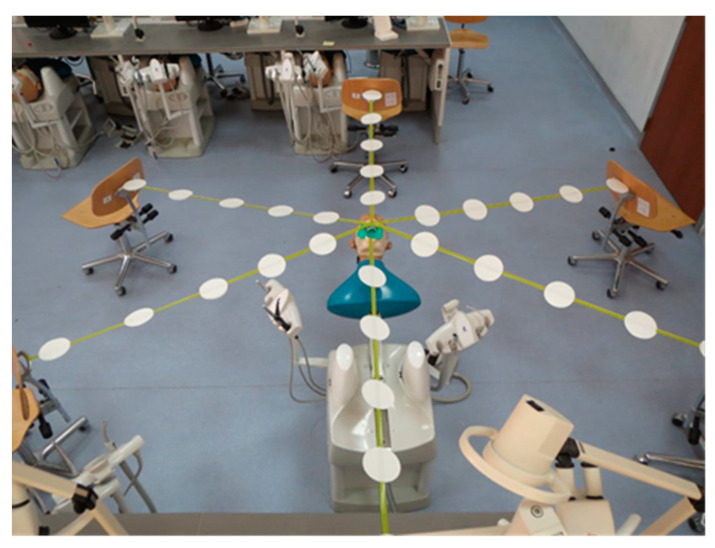
Distribution of filter papers around the dental manikin at different positions and distances.

The study was performed on a dental manikin in the dental simulation laboratory at the College of Dentistry, King Faisal University Al Ahsa. The manikin was set to a reclined position to simulate the clinical operatory position of the patient for dental restorative procedures. Rubber dam isolation was achieved for the upper anterior quadrant from tooth #13 to #23, as shown in Figure 1. Adhesive tape was placed in six different directions from the head of the manikin at 2, 4, 6, 8, 10, and 12 o’clock positions (Figure 2). Grade 1 qualitative cotton cellulose filter paper (Whatman; Maidstone, England) was attached on adhesive tape at a distance of 12-inches from each other up to 60-inches around the dental training manikin. Filter papers were also placed at certain positions of the operator and assistant such as the chest, head, forearms, upper leg, and inside facemask.

**Figure 3:**
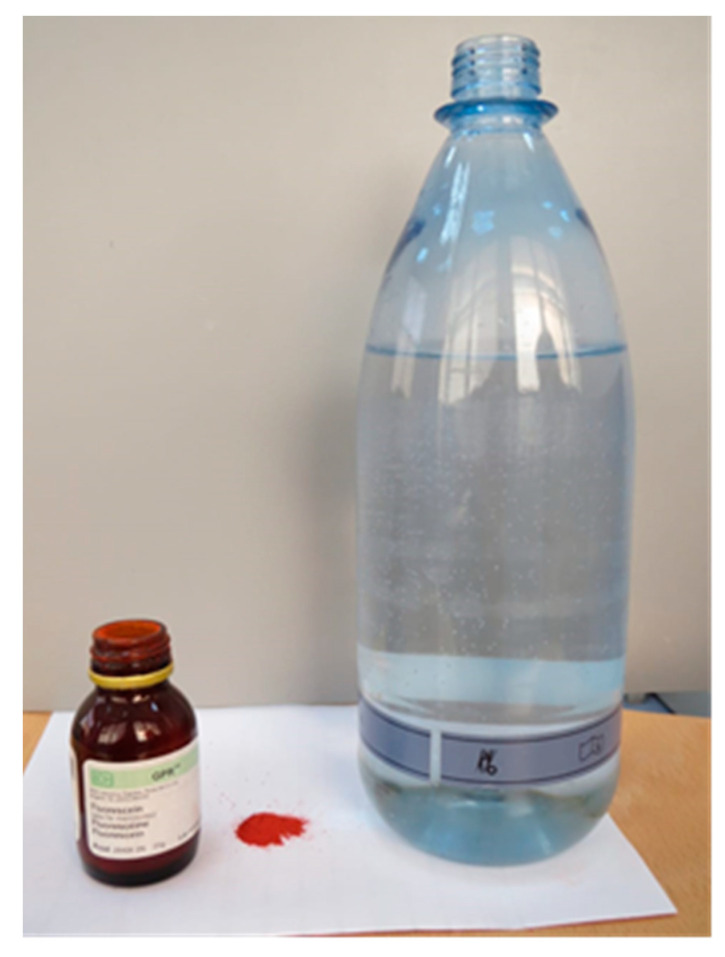
Dispersion of the ultra-filtrate containing the fluorescent dye.

One gram of ultra-filtrate containing fluorescent dye (Fluorescein, BDH, Poole, England) was mixed with one liter of water and filtered (Figure 3). This mixture was filled in a reservoir water bottle attached to the dental manikin.

**Figure 4:**
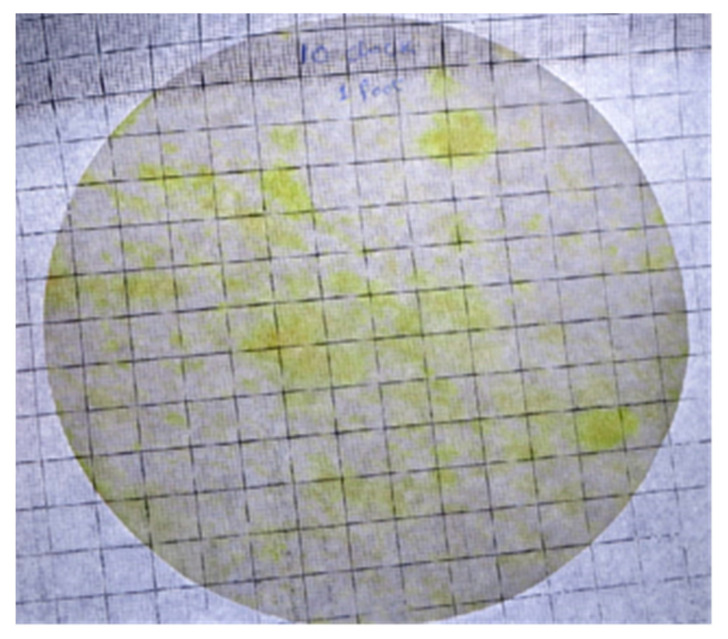
Filter paper disc with a transparent grid to count the contamination area.

Class V cavity preparation was done by the principal investigator in a specific time of 3 min on tooth #11 using a two-hole high-speed handpiece (PANA MAX, NSK, Tochigi, Japan) then on the next day, Class V cavity preparation was performed on tooth #21 by a four-hole handpiece (KAVO, Biberach, Germany). High volume suction attached to the dental manikin (DSEplus Type 5193, KAVO, Warthausen, Germany) was used with a vented tip throughout the cavity preparation, which was standardized by testing the liquid uptake of 1 L per minute. Immediately after cavity preparation, the first filter paper discs were replaced with new ones in all positions. The second set of filter papers were removed after 30 min. The splatter and aerosol contaminated area on the filter papers were calculated by using transparent grids containing a 1 cm^2^ box (Figure 4). Each filter paper covered 184 boxes maximum. Even a small amount in a square box was taken as a positive finding.

### Statistical Analysis

Data were analyzed using SPSS version 23.0. Mean and standard deviation was calculated for aerosol and splatter produced during cavity preparation at different positions and distances. An independent *t*-test was used to compare differences in the aerosol and splatter produced using two holes and four holes handpiece. Differences in the amount of splatter produced immediately after cavity preparation and 30 min after cavity preparation were assessed using a paired *t*-test. A *p*-value ≤ 0.05 was considered statistically significant.

## 3. Results

Immediately after the cavity preparation, the greatest amount of splatter of 74 cm^2^ and 70 cm^2^ were recorded using the two- and four-hole handpiece, respectively, at the 10 o’clock position and a distance of 12 inches. The amount of splatter decreased with an increase in distance and was found to be the lowest at a maximum distance of 60 inches away from the patient (Table 1). Most splatters were produced at the 10 o’clock position followed by 2 o’clock, 12 o’clock, 9 o’clock, 6 o’clock, and least at the 4 o’clock position.

The assessment of the contaminated surface area on the body revealed that the operator area was more contaminated (254 cm^2^) compared to the assistant area (197 cm^2^). The operator’s right hand and the assistant’s left hand were most prone to contamination by the splatter during cavity preparation, followed by the head, chest, and the least contamination was inside the mask for both the operator and assistant (Table 2).

Comparing the two and four-hole handpieces, no significant difference was found in the mean amount of aerosol and splatter produced by both handpieces (Table 3). The amount of aerosol and splatter produced was greatly reduced after 30 min of cavity preparation and only a meager amount was found at a distance of 12 inches and almost no splatter at farther distances (Table 4). Statistically, significant differences were found in the amount of aerosol and splatter produced at distances of 12, 24, and 36 inches immediately after cavity preparation and 30 min after cavity preparation, regardless of the type of handpiece used.

## 4. Discussion

This study was conducted to evaluate the amount and duration of aerosol and splatter produced during cavity preparation using a two-hole and four-hole high-speed handpiece, at various positions and distances. The result of the current study showed no statistically significant difference in the mean amount of aerosol and splatter produced by both handpieces, however, a statistically significant difference was found in the amount of aerosol and splatter produced at distances of 12, 24, and 36 inches immediately after cavity preparation and 30 min after cavity preparation, regardless of the type of handpiece used.

In this study, maximum splatter and aerosol produced immediately after the procedure were found at the operator zone, followed by the assistant zone. This finding contradicts the previous study in which investigators found more splatter in the assistant zone compared to the operator zone [4]. This difference in findings could be due to different types of dental procedures used for the generation of aerosol and splatter. Furthermore, higher splatter was found on the right arm of the operator and left arm of the assistant followed by the head, chest, and inside the face mask. This result is partially in line with the previous study [4] in which the operator’s right arm and assistant’s left arm were the highest spread area, while the chest was the second most affected area.

Indubitably, various contagious diseases such as HIV and HBV are transmitted through blood, saliva, and the gingival fluid of patients with known or unknown infection; therefore, it is wise to consider all patients potentially infective, irrespective of their medical history [20,21]. Additionally, arm protection is necessary as minor skin scratches are often unrecognized and can be exposed to blood and saliva splatter during the procedure [22]. However, the risk from exposure to impaired skin or mucous membrane is far less likely than that from parenteral contact such as needle prick injury [23].

Recently, COVID-19 infection has received great attention due to the easy transmission through the respiratory route. Ahmed et al. [14] reported fear and anxiety among dentists from different countries of acquiring COVID-19 infection during practice and unintentionally causing harm to their families. Considering the current situation, this study was designed to follow the recommendations from the ADA (American Dental Association) interim guidance to curb the transmission of COVID-19 [24]. The measures included were the use of a rubber dam, four-handed dentistry, and high volume suction. In this study, the penetration of fluorescent dye on the filter paper inside the facemask was found, regardless of the type of handpiece. This result was similar to the previous study in [23], despite the use of a three-layered facemask in our study compared to a one-layered facemask. This finding further endorsed the importance of using a facemask and other protective barriers such as a face shield and external oral suction device to reduce the chance of spreading infection, especially during the time of COVID-19.

The two-hole handpiece is also called Borden’s handpiece, after its successful development by Dr. John Borden’s in 1950. The turbine and body are the main components of the air-driven handpiece. Stainless steel and brass are the most common materials used to manufacture handpieces. Stainless steel handpieces are heavier and expensive than brass handpieces. However, titanium-based handpieces are preferable nowadays by clinicians and the manufacturer due to their lighter weight and greater resistance to repeated sterilization cycles compared to stainless steel [9]. In the present study, although no significant difference was found between the two-hole and four-hole handpiece, however, overall splatter was noticed more in the two-hole handpiece at all positions and distances. A possible explanation for this finding in the two-hole handpiece could be related to its working mechanism, during handpiece working, air traps in the head lead to the continuous rotation of the bur, even after the release of the air-water spray foot pedal; this phenomenon is called the coast speed. With time, manufacturers developed three-hole handpieces to reduce coast speed in which trapped air at the head of the handpiece easily comes out from the third hole that acts as a vent, and thus the bur comes to a pause at the earliest [9,10]. Likewise, the four-hole handpiece has been marketed with an additional hole for fiber optics.

It has been noticed that the whole circumference around the manikin showed splatter contamination for up to 36 inches immediately after the procedure except at the 4 o’clock position with both handpieces and 12 o’clock with the four-hole handpiece. This result is similar to Llandro et al. [25] in which they found a one-meter (39 inches) circumferential spread of splatter in a positive control group (crown preparation with high-speed handpiece). In contrast, Veena et al. [4] found the circumferential spread of splatter up to 12-inches around the dental manikin; which may be due to differences in the procedure (ultrasonic scaling) and technique used for the study.

Bennet et al. [26] reported that aerosol remains in the practice for around 10 to 30 min following scaling. This finding conforms to our study in which the amount of aerosol was found mostly at a distance of 12-inches after 30 min and almost no aerosol at farther distances, although a significant reduction was noticed after 30 min compared to the time of immediate cavity preparation. Therefore, to reduce the risk of contamination from airborne pathogens, especially in this era of the COVID-19 pandemic, it is recommended that practitioners keep wearing their personal protective barriers after the completion of the procedure for a certain period [27].

Considering the findings of the current study, aerosol and splatter can spread up to 36 inches; therefore it is desirable to make an arbitrary “red zone” around the dental unit. This red zone will require thorough cleaning and disinfection by antiviral or antimicrobial disinfectant after every patient. No one should be allowed to enter the red zone during the procedure, except for the dentist and the assistant.

Clinic ventilation plays a major role in mitigating the spread of airborne infections. Sarapultseva et al. [28] reported that the use of aspirating systems installed with HEPA (high-efficiency particulate air) filters can help to evacuate and dissipate aerosols into a specialized area away from the dental practice, thus providing a safe ambiance to the dental staff, especially in the era of the COVID-19 pandemic. However, these are expensive equipment and are not cost-effective. Another reasonable alternative in hand is external high volume suction. In a high-volume evacuator, the use of a large-bore tip diameter (8 mm and above) can efficiently reduce aerosol and splatter up to 93–96% by removing air at 100 cubic feet per minute [29].

This study has some limitations that include the use of liquid volume uptake to measure suction flow due to the unavailability of a flowcheck device. Moreover, the concentration of splatter was not assessed and even a single drop was counted as positive in measuring grid squares. Since the tooth preparation was done only on anterior teeth by the high-speed handpiece, the results should be interpreted cautiously and cannot be generalized, as the results might be different for posterior teeth and with the use of some other devices such as a low-speed handpiece or ultrasonic scaler. Finally, a complete study was conducted on the dental manikin, which does not have saliva and blood, and hence may not produce a real-life work scenario.

## 5. Conclusions

This study emphasizes that the dissemination of contaminated aerosol and splatter after cavity preparation remains a major threat to dental staff in the era of the COVID-19 pandemic. Therefore, it is advisable to use surgical disposable gowns, caps, face shields, or safety glasses, and refrain from removing the personal protective barriers immediately after the procedure within the vicinity of the dental practice. The use of other adjuncts such as high volume suction to reduce the spread of aerosol and splatter is also recommended.

## Figures and Tables

**Table 1:** Distribution of aerosol and splatter at different positions and distances by the two- and four-hole handpiece.

Distance	Contaminated Surface Area (cm^2^)											
	Two-Hole Handpiece						Four-Hole Handpiece					
	12 o’clock	2 o’clock	4 o’clock	6 o’clock	8 o’clock	10 o’clock	12 o’clock	2 o’clock	4 o’clock	6 o’clock	8 o’clock	10 o’clock
**12 inches**	63	71	41	55	60	74	59	60	36	48	55	70
**24 inches**	49	58	19	38	28	53	40	54	10	29	30	45
**36 inches**	15	30	-	19	13	22	-	23	-	11	12	27
**48 inches**	-	11	-	4	-	10	-	5	-	-	-	7
**60 inches**	-	4	-	-	-	3	-	-	-	-	-	-
**Mean**	25.4	34.8	12	23.2	20.2	32.4	19.8	28.4	9.2	17.6	19.4	29.8
**St. Dev**	29.02	29.09	18.17	23.2	25.06	30.12	27.93	27.55	15.59	20.71	23.38	28.56

**Table 2:** Collection of aerosol and splatter on different body parts of the operator and assistant.

Body Part	Contaminated Surface Area (cm^2^) Operator	Contaminated Surface Area (cm^2^) Assistant
**Head**	67	52
**Chest**	55	39
**Right arm**	91	19
**Left arm**	34	85
**Inside mask**	7	2
**Total**	254	197

**Table 3:** Comparison of the mean aerosol and splatter produced by two-hole and four-hole handpiece.

Distance	*p*-Value	Mean Difference	Std. Error Difference
**12 Inches**	0.398	6.00000	6.79379
**24 Inches**	0.500	6.16667	8.81318
**36 Inches**	0.498	4.33333	6.16532
**48 Inches**	0.401	2.16667	2.46869
**60 Inches**	0.150	1.16667	0.74907

**Table 4:** Comparison of the mean aerosol and splatter produced immediately after cavity preparation and 30 min after cavity preparation using a two-hole and four-hole handpiece.

Handpieces	Distance	Time	Mean	St. Deviation	*p*-Value
**Two holes**	12 inches	Post filling	60.6667	11.91078	0.000
		After 30 min	10.17	5.707	
	24 inches	Post filling	40.8333	15.22388	0.001
		After 30 min	0.50	1.225	
	36 inches	Post filling	16.5000	10.05485	0.010
		After 30 min	0.00	0.000	
	48 inches	Post filling	4.1667	5.15429	0.105
		After 30 min	0.00	0.000	
	60 inches	Post filling	1.1667	1.83485	0.180
		After 30 min	0.00	0.000	
**Four holes**	12 inches	Post filling	54.6667	11.62182	0.000
		After 30 min	3.17	3.817	
	24 inches	Post filling	34.6667	15.30577	0.003
		After 30 min	0.00	0.000	
	36 inches	Post filling	12.1667	11.26795	0.046
		After 30 min	0.00	0.000	
	48 inches	Post filling	2.0000	3.16228	0.182
		After 30 min	0.00	0.000	
	60 inches	Post filling	0.0000	0.00000	-
		After 30 min	0.00	0.000	

## Data Availability

Not applicable.
